# Association between spot urinary sodium-to-potassium ratio and blood pressure among Chinese adults aged 18–69 years: the SMASH study

**DOI:** 10.3389/fnut.2024.1383243

**Published:** 2024-06-05

**Authors:** Chunxiao Xu, Jing Dong, Danru Liu, Jianwei Xu, Bingyin Zhang, Zilong Lu, Linhong Wang, Junli Tang, Xiaochang Zhang, Jie Ren, Xiaohui Yu, Rui Guo, Xiaolei Guo, Jing Wu, Jixiang Ma

**Affiliations:** ^1^Shandong Center for Disease Control and Prevention, Jinan, Shandong, China; ^2^National Center for Chronic and Noncommunicable Disease Control and Prevention, Chinese Center for Disease Control and Prevention, Beijing, China; ^3^School of Public Health, Shandong University, Jinan, Shandong, China

**Keywords:** sodium-to-potassium ratio, spot urine, hypertension, blood pressure, Chinese

## Abstract

**Background:**

Excessive sodium and low potassium intake are involved in the development of hypertension. Growing evidence showed that the sodium-to-potassium ratio (Na/K) was significantly associated with blood pressure (BP). However, studies on the dose-response relationship of spot urinary Na/K ratio with hypertension and BP in the general population are scarce, especially in the Chinese population.

**Materials and methods:**

Data from the post-intervention survey of the Shandong Ministry of Health Action on Salt and Hypertension (SMASH) project was analyzed. Associations between Na/K molar ratio and hypertension prevalence and between Na/K molar ratio and BP indices were analyzed using multivariable logistic and linear regression, respectively, followed by subgroup analysis and interaction analysis. The restricted cubic spline model was used to explore the dose–response relationship. Informed by existing literature, we adjusted for potential confounding factors, including temperature and renal function, to assess the association and dose–response relationship.

**Results:**

There was a non-linear positive association between Na/K and hypertension (OR:1.09, 95%CI: 1.08–1.11) and a linear positive association between Na/K and systolic BP, diastolic BP, and mean arterial pressure (β 0.53, 95%CI: 0.45–0.60; β 0.36, 95%CI: 0.31–0.41; and β 0.42, 95%CI: 0.36–0.47, respectively). The association was stronger in individuals with hypertension, female patients, those in the 50–59-year age group, and those who were obese. Environmental temperatures had little impact on associations.

**Conclusion:**

Our findings provide further evidence that the spot urinary Na/K ratio is a simple, useful, and convenient indicator for monitoring salt reduction and potassium increase, which could be used in clinical and public health practices.

## 1 Introduction

Cardiovascular disease (CVD) is a major public health concern and remains the leading cause of global mortality (1). Excessive sodium intake is considered a significant risk factor for CVD, contributing to ~1.89 million CVD deaths in 2019 (2). The World Health Organization (WHO) listed salt reduction as one of the five priority intervention strategies for non-communicable disease prevention in 2007 (3) and proposed a global target of a 30% reduction in sodium intake by 2025 in 2013 (4). Nevertheless, none of the WHO Member States has yet reached the target (5). Evidence suggests that a reduction in dietary sodium intake alone is challenging to achieve (6). Potassium ions, antagonistically and synergistically interacting with sodium ions to maintain homeostasis, are important for life. Growing evidence indicates that high potassium intake can reduce blood pressure (BP), and this BP-lowering effect may attenuate the BP-raising effect of excessive sodium intake (7–10). Therefore, increasing potassium intake with salt reduction may be more beneficial than salt reduction alone.

China is one of the countries with the highest daily salt consumption, with an average salt intake per capita of 9.3 g/day (11), nearly twice the WHO recommendation (12). Excessive salt intake was one of the top three risk factors for death and life-years lost in China, accounting for more than 5% of disability-adjusted life years (DALYs) (13). In addition, the average potassium intake is 1,417 mg/day, < ½ of the recommended amount of 3,500 mg/day (14). Shandong province, the second most populous province in China, has the highest death rate from hypertension and the second highest death rate from CVD in the country (15). In 2011, the Shandong Ministry of Health Action on Salt and Hypertension (SMASH) project was launched by the People's Government of Shandong Province and the National Health Commission of the People's Republic of China (formerly the Ministry of Health) (16). After 5 years of large-scale population salt reduction action, urinary sodium excretion decreased to 4,013 mg/day and potassium excretion increased to 1,850 mg/day (17). While effective progress has been demonstrated, a large gap still exists between the current intake and the recommended intake of sodium and potassium.

To improve the dietary consumption of high sodium and low potassium, restricting sodium intake and simultaneously increasing potassium intake are urgently needed in China. Currently, the sodium-to-potassium ratio has been regarded as a better monitoring indicator than the ion alone (17–21). Clarifying the relationship between the urinary Na/K ratio and BP will aid in developing effective public health strategies. Numerous studies based on 24-h urine collection have shown that a higher urinary Na/K ratio is associated with higher BP levels, regardless of the relationship between sodium intake and BP (21–26). In our previous research, we found an insignificant association between sodium intake and BP but a positive association between 24-h urinary Na/K ratio and BP, which was only observed in overweight/obese individuals (27). However, the evidence for the relationship between spot urinary Na/K ratio and BP or hypertension is limited in China, especially in different population subgroups. We analyzed data from the post-intervention survey of SMASH—a provincial representative cross-sectional survey to address this research gap. We aimed to examine the association between spot urinary Na/K ratio and BP or hypertension, assess variations among population subgroups, and identify the key populations for tailored intervention.

## 2 Materials and methods

### 2.1 Study design

Data for this study were obtained from the post-intervention survey of the SMASH program—a cross-sectional survey conducted from 20th June 2016 to 29th August 2016. This survey used a multistage stratified sampling method to select a provincially representative sample of the adult population aged 18–69 years. A total of 16,490 eligible participants from 156 villages or communities in 8 urban districts and 12 rural counties were investigated through face-to-face interviews to administer questionnaires and collect spot urine (SU) samples. A subsample of 2,019 participants was selected from 52 randomly chosen villages or communities across the aforementioned districts and counties, and they were asked to collect 24-h urine samples. Detailed methods have been published elsewhere (28). The ethical committee of the Shandong Center for Disease Control and Prevention approved the post-intervention survey. All respondents provided written informed consent.

### 2.2 Urine collection and estimation of 24-h urinary sodium and potassium excretion

Trained health professionals informed participants of the detailed procedure for SU and 24-h urine collection. All participants were asked to collect a fasting morning urine sample at home and take the collection tube to the survey site. A total of 2019 subjects were provided a 5 L urine container and a 2 L urine container to collect 24-h urine samples. The participants collecting 24-h urine samples were instructed to empty their bladders at the survey site and record the time of their morning's micturition as the start time of 24-h urine collection. All urine samples over the following 24-h were collected. Trained health professionals recorded the start and finish duration of 24-h urine collections and asked for all missed collections carefully. The total volume was measured and recorded. If the urine volume was <0.5 L or more than two omissions were documented, urine collections were discarded due to incompletion. Finally, a 5-ml urine aliquot of SU or 24-h urine sample was extracted and frozen at −20°C for further analysis.

All urine aliquots were shipped to a certified laboratory for further analysis. Urinary sodium and potassium were detected by the ion-selective electrode method, whereas urinary creatinine was measured with the enzyme method. To ensure the accuracy and reliability of laboratory test results, standard quality control samples at both high and low concentration levels were employed for quality control purposes. The coefficient of variation (CV) for the low and high concentration levels of sodium, potassium, and creatinine resulted in values of 1.97 and 1.17%, 1.18 and 1.95%, and 2.41 and 2.88%, respectively. All of these values fell within the maximum allowable CV specified by the Ministry of Health's industry standards, which are 2.8% for sodium, 3.43% for potassium, and 5% for creatinine.

Urine samples were regarded as complete when 24-h urinary creatinine excretion was in the sex-specific mean ± 2 standard deviation (SD). Estimated excretion values of sodium and potassium were calculated using the Tanaka equation-based SU sample. The Tanaka equation is as follows (29): (1) predicted 24-h urinary creatinine excretion (PrUCr_24-*h*_, mg/day) = 14.89 × weight (kg) + 16.14 × height (cm) – 2.04 × age (years) – 2,244.45; (2) estimated 24-h urinary sodium excretion (24-h eUNa, mmol/day) = 21.98 × (spot urinary sodium (mmol/L)/spot urinary creatinine (mg/L) × PrUCr_24-*h*_ [mg/day)]^0.392^; and (3) estimated 24-h urinary potassium excretion (24-h eUK, mmol/day) = 7.59 × (spot urinary potassium (mmol/L)/spot urinary creatinine (mg/L) × PrUCr_24-*h*_ [mg/day)]^0.431^.

### 2.3 Collection and definition of other variables

Sociodemographic information, history of non-communicable diseases, knowledge, attitudes, and behaviors (KABs) related to dietary sodium intake and hypertension, and lifestyle information were obtained using a questionnaire. We identified three primary KAB indicators in the present study, including “As salt intake decreases, so does blood pressure,” “Approval of a low salt diet,” and “Have taken action to reduce dietary salt intake.” These indicators were defined based on positive responses, indicating agreement or the enactment of the stated behavior. Regular physical exercise was defined as any physical activity performed at least three times a week and lasting 30 min or more each time.

A fasting blood specimen was collected for the measurement of glucose, lipid profiles, and creatinine. Glomerular filtration rate (eGFR) was estimated from blood creatinine by the chronic kidney disease epidemiology collaboration (CKD–EPI) equation (30). Other measurements, including height, weight, waist circumference, and blood pressure, were also obtained by trained health professionals. BP was measured three times using a validated electronic sphygmomanometer (HEM-7071, Omron Corporation, Dalian, China) at 1-min intervals, and the average was used. All participants are classified into three groups based on their BP levels: the normal group, the prehypertension group, and the hypertension group. Hypertension was defined as the average systolic blood pressure (SBP) ≥ 140 mmHg and/or diastolic blood pressure (DBP) ≥ 90 mmHg, or self-reported diagnosis of hypertension in hospitals at the township level and above, or taking antihypertensive medicine in the last 2 weeks. Prehypertension was defined as the SBP of 120–139 mmHg and/or the DBP of 80–89 mmHg, and normotensives were participants with a BP <120/80 mmHg. We also calculated mean arterial pressure (MAP) by using (SBP + 2^*^DBP)/3.

The season of urine collection can potentially affect the urinary excretion of cations due to varying sweat excretion rates throughout different seasons. To mitigate this bias, we have also collected data on environmental temperature for adjustment. Environmental temperature data from the day before the on-site investigation were obtained from the National Meteorological Science Data Sharing Center.

### 2.4 Statistical analysis

We used R software (version 4.3.1) to analyze the data. A *p*-value of <0.05 was considered to be statistically significant.

We first excluded participants without spot urine specimens (*n* = 310). Outliers of Na/K and estimated 24-h urinary sodium and potassium excretion [exceeding three times the interquartile range (IQR)], were also excluded (*n* = 134). After the exclusion criteria were applied, 16,046 participants were included in the analytic sample. Categorical variables were expressed in the form of percentages. Normally distributed variables were described as mean ± standard deviation, while non-normally distributed variables were presented as median (IQR). Differences between groups were compared using the *t*-test, the χ^2^-test, and the non-parametric test.

Multivariate logistic regression models and linear regression models were used to explore the associations of Na/K with hypertension prevalence and blood pressure levels, respectively. Spot urinary Na/K was analyzed as a categorical variable (grouped by quartiles) and as a continuous variable in the above analyses. In these multivariate analyses, variables including age, gender, region, residence, education, occupation, income level, body mass index (BMI), regular physical exercise, smoking, alcohol consumption, history of diabetes, history of stroke, history of coronary heart disease (CHD), and KABs of dietary sodium intake and hypertension, eGFR and average temperature were adjusted. Antihypertensive medication use was also adjusted in the linear regression models and in the analysis specific to participants in the hypertension groups. Then, we used restricted cubic spline (RCS) models to clarify the dose–response relationship between the Na/K ratio and hypertension prevalence or blood pressure. Moreover, further subgroup analysis was performed to explore the interactive effects of age, gender, BMI, smoking, alcohol use, and physical exercise on the Na/K ratio.

We also carried out sensitivity analysis using the 24-h urinary indices. Among the 2019 participants who collected 24-h urine samples, 344 were excluded because of incomplete collection, and four were excluded due to a lack of spot urine specimens. To evaluate the potential influence of different urine specimens on studied associations, we performed logistic regression, linear regression, and the RCS model in the remaining 1,671 participants who collected both 24-h urine and spot urine samples. Other sensitivity analyses were also conducted as follows: (1) excluding participants who used anti-hypertension medicines; (2) excluding participants with stroke or CHD; (3) removing alcohol use from adjusted variables; (4) removing regular physical exercise from adjusted variables; (5) removing occupation and income levels from adjusted variables; and (6) removing KABs of salt and hypertension from adjusted variables.

## 3 Results

### 3.1 Descriptive analysis of the studied population

Among 16,046 (50.33% female) participants, the mean age was 42.21 ± 13.37 years. More than 30% of participants had prehypertension, and 20.11% of participants had hypertension. The age and gender distribution, residence, region, education, occupation, income, lifestyle status, prevalence of diabetes, stroke and CHD, and BMI were all significantly different in the different BP categories (*P* < 0.05; Table 1). Participants with hypertension had better salt reduction knowledge and behavior. Both estimated excretion from spot urine specimens and measured excretion from 24-h urine samples showed that participants with hypertension had significantly higher Na/K molar ratio and sodium excretion.

**Table 1:** Characteristics of the 16,046 participants in the SMASH post-intervention survey.

**Variables**	**All (*n* = 16,046)**	**Normal (*n* = 7,597)**	**Prehypertension (*n* = 4,902)**	**Hypertension (*n* = 3,547)**	***P*-value**
Age (years, mean ± SD)	42.21 ± 13.37	38.19 ± 12.66	42.63 ± 13.08	50.24 ± 11.38	<0.0001
Age group [*n* (%)]					<0.0001
18–29	3,717 (23.16)	2,433 (32.03)	1,052 (21.46)	232 (6.54)	
30–39	3,148 (19.62)	1,836 (24.17)	926 (18.89)	386 (10.88)	
40–49	4,138 (25.79)	1,810 (23.83)	1,370 (27.95)	958 (27.01)	
50–59	3,122 (19.46)	1,019 (13.41)	984 (20.07)	1,119 (31.55)	
60–69	1,921 (11.97)	499 (6.57)	570 (11.63)	852 (24.02)	
Gender [*n* (%)]					<0.0001
Man	7,970 (49.67)	3,060 (40.28)	3,013 (61.46)	1,897 (53.48)	
Woman	8,076 (50.33)	4,537 (59.72)	1,889 (38.54)	1,650 (46.52)	
Ethnicity [*n* (%)]					0.304
Han	16,000 (99.71)	7,570 (99.64)	4,891 (99.78)	3,539 (99.77)	
Others	46 (0.29)	27 (0.36)	11 (0.22)	8 (0.23)	
Residence [*n* (%)]					0.0001
Urban	4,971 (30.98)	2,478 (32.62)	1,449 (29.56)	1,044 (29.43)	
Rural	11,075 (69.02)	5,119 (67.38)	3,453 (70.44)	2,503 (70.57)	
Region [*n* (%)]					<0.0001
East	3,995 (24.90)	1,645 (21.65)	1,397 (28.5)	953 (26.87)	
Middle and South	6,151 (38.33)	3,015 (39.69)	1,909 (38.94)	1,227 (34.59)	
Northwest	5,900 (36.77)	2,937 (38.66)	1,596 (32.56)	1,367 (38.54)	
Education [*n* (%)]					<0.0001
Primary, middle school, and under	11,762 (73.30)	5,248 (69.09)	3,639 (74.24)	2,875 (81.05)	
High school and above	4,283 (26.69)	2,348 (30.91)	1,263 (25.76)	672 (18.95)	
Occupation [*n* (%)]					<0.0001
(Hard physical work) Farmer/ Peasant/Manual worker	9,272 (57.78)	4,002 (52.69)	3,002 (61.24)	2,268 (63.94)	
(Light physical work) Service/Administrative/Technical/ Professionals/Others	5,921 (36.90)	3,246 (42.74)	1,660 (33.86)	1,015 (28.62)	
Underemployment/Retired	851 (5.30)	347 (4.57)	240 (4.90)	264 (7.44)	
Annual household income [*n* (%)]					<0.0001
First tertile	5,336 (34.19)	2,343 (31.79)	1,604 (33.56)	1,389 (40.17)	
Second tertile	5,119 (32.80)	2,481 (33.66)	1,584 (33.14)	1,054 (30.48)	
Third tertile	5,154 (33.02)	2,547 (34.55)	1,592 (33.31)	1,015 (29.35)	
Lifestyle status [*n* (%)]					
Smoking	4,525 (28.21)	1,779 (23.43)	1,642 (33.51)	1,104 (31.13)	<0.0001
Alcohol consumption	5,392 (33.62)	2,060 (27.13)	1,962 (40.04)	1,370 (38.64)	<0.0001
Regular physical exercise	3,025 (18.85)	1,274 (16.77)	918 (18.73)	833 (23.48)	<0.0001
Disease status [*n* (%)]					
Diabetes	1,325 (8.26)	259 (3.41)	429 (8.75)	637 (17.96)	<0.0001
Stroke	189 (1.18)	22 (0.29)	32 (0.65)	135 (3.81)	<0.0001
Coronary heart disease	411 (2.56)	93 (1.22)	83 (1.69)	235 (6.63)	<0.0001
KABs of salt and hypertension					
As salt intake decreases, so does blood pressure	10,995 (68.53)	5,051 (66.50)	3,341 (68.17)	2,603 (73.39)	<0.0001
Approval of a low-salt diet	14,773 (92.08)	6,978 (91.88)	4,514 (92.10)	3,281 (92.50)	0.523
Has taken action to reduce dietary salt intake	10,167 (63.37)	4,746 (62.49)	3,042 (62.07)	2,379 (67.07)	<0.0001
BMI (kg/m^2^, mean ± SD)	25.18 ± 4.12	23.76 ± 3.62	25.76 ± 3.88	27.43 ± 4.27	<0.0001
SBP (mmHg, mean ± SD)	120.85 ± 17.19	107.55 ± 7.70	125.81 ± 6.38	142.51 ± 16.50	<0.0001
DBP (mmHg, mean ± SD)	76.99 ± 11.41	68.79 ± 6.14	79.70 ± 5.81	90.79 ± 10.82	<0.0001
MAP (mmHg, mean ± SD)	91.61 ± 12.76	81.71 ± 6.01	95.07 ± 4.66	108.03 ± 11.54	<0.0001
Spot urine indices					
Sodium concentration (mmol/L, mean ± SD)	137.89 ± 62.27	133.83 ± 62.28	141.08 ± 62.32	142.2 ± 61.66	<0.0001
Potassium concentration (mmol/L, mean ± SD)	35.00 ± 23.68	36.05 ± 24.25	35.38 ± 23.42	32.26 ± 22.56	<0.0001
Creatinine concentration (mmol/L, mean ± SD)	10.20 ± 6.05	10.62 ± 6.16	10.59 ± 6.17	8.76 ± 5.40	<0.0001
Sodium/potassium ratio [median (IQR)]	4.51 (2.93–6.70)	4.19 (2.75–6.27)	4.53 (3.04–6.66)	5.27 (3.28–7.63)	<0.0001
Estimated sodium excretion^a^ (*g/day*, mean ± SD)	3.83 ± 0.90	3.63 ± 0.82	3.87 ± 0.87	4.20 ± 0.99	<0.0001
Estimated potassium excretion^a^ (*g/day*, mean ± SD)	1.45 ± 0.33	1.42 ± 0.33	1.47 ± 0.34	1.52 ± 0.33	<0.0001
Predicted creatinine excretion^b^ (g/day, mean ± SD)	1.31 ± 0.30	1.24 ± 0.26	1.37 ± 0.31	1.36 ± 0.32	<0.0001
eGFR^c^ (ml/min per 1.73 m^2^, mean ± SD)	106.37 ± 13.42	109.72 ± 12.43	106.1 ± 12.96	99.56 ± 13.45	<0.0001
24-h urine indices^*^					
Urine volume (L)	1,545.19 ± 599.96	1,532.38 ± 585.85	1,506.27 ± 596.26	1,614.21 ± 624.81	0.019
Sodium concentration (mmol/L, mean ± SD)	123.81 ± 56.97	118.8 ± 54.96	129.93 ± 59.54	125.67 ± 56.79	0.003
Potassium concentration (mmol/L, mean ± SD)	33.65 ± 17.75	33.15 ± 17.48	35.57 ± 17.86	32.27 ± 17.95	0.013
Creatinine concentration (mmol/L, mean ± SD)	7.73 ± 3.89	7.53 ± 3.76	8.47 ± 4.12	7.22 ± 3.72	<0.0001
Sodium/potassium ratio [median (IQR)]	3.76 (2.68–5.30)	3.69 (2.67–5.04)	3.74 (2.68–5.25)	4.02 (2.74–6.03)	0.016
Measured sodium excretion (*g/day*, mean ± SD)	4.06 ± 1.82	3.85 ± 1.66	4.13 ± 1.78	4.35 ± 2.08	<0.0001
Measured potassium excretion (*g/day*, mean ± SD)	1.84 ± 0.85	1.81 ± 0.83	1.89 ± 0.81	1.85 ± 0.92	0.262
Measured creatinine excretion (g/day, mean ± SD)	1.19 ± 0.40	1.15 ± 0.38	1.27 ± 0.41	1.17 ± 0.43	<0.0001

BMI, body mass index; SBP, systolic blood pressure; DBP, diastolic blood pressure; MAP, mean arterial pressure; eGFR, estimated glomerular filtration rate; IQR, interquartile range.

^a^Estimated excretion was calculated using the Tanaka equation from a fasting morning urine specimen.

^b^Estimated creatinine excretion was calculated using a formula that was also used in the Tanaka equation to estimate sodium and potassium excretion.

^c^Estimated from blood creatinine by the CKD-EPI formula.

^*^Results for the subsample with 24-h urine collection (n = 1,671).

### 3.2 Distribution of the primary spot urinary Na/K molar ratio in the studied population

The distribution of spot urinary Na/K ratio was summarized in Supplementary Table 1. Significant differences in the Na/K ratio among different genders, regions, educations, and occupations were observed (*P* < 0.05). The spot urine Na/K rose significantly with age and BMI, respectively (*P* < 0.05). Participants who smoked, consumed alcohol, exercised irregularly, had high blood pressure, or had a history of stroke exhibited a higher Na/K ratio compared to their counterparts (*P* < 0.05). Respondents who were aware of the relationship between salt intake and blood pressure favored low salt diets and had already taken action toward salt reduction had a lower Na/K ratio (*P* < 0.05).

### 3.3 Spot urinary Na/K ratio with hypertension prevalence and blood pressure levels

As shown in Tables 2, 3, the spot urinary Na/K ratio was positively associated with the risk of hypertension and blood pressure levels. In the fully adjusted model, the third and fourth quartile groups of the Na/K ratio had a higher prevalence of hypertension than the first quartile group (third: OR 1.22, 95% CI 1.08–1.39; fourth: OR 1.78, 95% CI 1.57–2.01), and there was a linear trend (*P* < 0.001; Table 2). The third and fourth quartile groups of the Na/K ratio were significantly correlated with SBP (third: β 2.21, 95% CI 1.57–2.84; and fourth: β 3.88, 95% CI 3.23–4.53) and MAP (third: β 1.63, 95% CI 1.16–2.10; and fourth: β 2.99, 95% CI 2.51–3.47) levels, while the highest quartile of the Na/K ratio had a significant correlation with DBP levels (β 2.55, 95% CI 2.11–2.98; Table 3). The linear trend across different quartiles was also significant (*P* for trend test <0.0001).

**Table 2:** Associations of Na/K (categorical variable) with the risk of hypertension.

**Outcome**	**Quartile of urinary sodium-to-potassium ratio**			
	**Q1 (<2.93)**	**Q2 (2.93–4.51)**	**Q3 (4.51–6.70)**	**Q4 (>6.70)**
**Hypertension**				
Crude^a^	1.00 (Ref.)	0.98 (0.87, 1.09)	1.38 (1.24, 1.54)^*^	1.93 (1.74, 2.14)^*^
Model 1^b^	1.00 (Ref.)	0.92 (0.81, 1.03)	1.28 (1.14, 1.43)^*^	1.89 (1.69, 2.11)^*^
Model 2^c^	1.00 (Ref.)	0.92 (0.81, 1.05)	1.25 (1.11, 1.42)^*^	1.83 (1.62, 2.08)^*^
Model 3^d^	1.00 (Ref.)	0.91 (0.80, 1.04)	1.22 (1.08, 1.39)^*^	1.78 (1.57, 2.01)^*^

Data are odds ratio (OR) and its 95% CI, ^*^P <0.05.

^a^Crude model adjusted for none.

^b^Model 1 included adjustment of gender and age.

^c^Model 2 included adjustment of gender, age, region, residence, education, occupation, income level, BMI, regular physical exercise, smoking, alcohol consumption, KABs of dietary sodium intake and hypertension, history of diabetes, history of stroke, history of CHD, and eGFR.

^d^Model 3: model 2 plus adjustment for average temperature.

**Table 3:** Associations of Na/K (categorical variable) with SBP, DBP, and MAP levels.

**Outcome**	**Quartile of urinary sodium-to-potassium ratio**			
	**Q1 (<2.93)**	**Q2 (2.93-4.51)**	**Q3 (4.51-6.70)**	**Q4 (>6.70)**
**SBP**				
Crude^a^	1.00 (Ref.)	0.97 (0.22, 1.72)	3.21 (2.47, 3.96)^*^	5.75 (5.01, 6.50)^*^
Model 1^b^	1.00 (Ref.)	0.52 (−0.17, 1.21)	2.21 (1.52, 2.89)^*^	4.62 (3.93, 5.31)^*^
Model 2^c^	1.00 (Ref.)	0.78 (0.15, 1.42)	2.27 (1.64, 2.91)^*^	3.97 (3.32, 4.61)^*^
Model 3^d^	1.00 (Ref.)	0.74 (0.11, 1.38)	2.21 (1.57, 2.84)^*^	3.88 (3.23, 4.53)^*^
**DBP**				
Crude^a^	1.00 (Ref.)	0.43 (−0.07, 0.92)	1.96 (1.46, 2.45)^*^	3.74 (3.25, 4.24)^*^
Model 1^b^	1.00 (Ref.)	0.21 (−0.27, 0.68)	1.46 (0.99, 1.93)	3.19 (2.71, 3.66)^*^
Model 2^c^	1.00 (Ref.)	0.29 (−0.14, 0.71)	1.43 (1.00, 1.85)^*^	2.66 (2.23, 3.09)^*^
Model 3^d^	1.00 (Ref.)	0.23 (−0.19, 0.66)	1.34 (0.91, 1.77)	2.55 (2.11, 2.98)^*^
**MAP**				
Crude^a^	1.00 (Ref.)	0.61 (0.06, 1.16)	2.38 (1.82, 2.93)^*^	4.41 (3.86, 4.96)^*^
Model 1^b^	1.00 (Ref.)	0.31 (−0.21, 0.83)	1.71 (1.19, 2.23)^*^	3.66 (3.15, 4.18)^*^
Model 2^c^	1.00 (Ref.)	0.45 (−0.01, 0.92)	1.71 (1.24, 2.18)^*^	3.10 (2.62, 3.57)^*^
Model 3^d^	1.00 (Ref.)	0.40 (−0.06,0.87)	1.63 (1.16,2.10)^*^	2.99 (2.51, 3.47)^*^

Data are regression coefficient (β) and its 95% CI, ^*^P <0.05.

^a^Crude model adjusted for none.

^b^Model 1 included adjustment of gender and age.

^c^Model 2 included adjustment of gender, age, region, residence, education, occupation, income level, BMI, regular physical exercise, smoking, alcohol consumption, KABs of dietary sodium intake and hypertension, history of diabetes, history of stroke, history of CHD, eGFR, and use of antihypertensive drugs.

^d^Model 3: model 2 plus adjustment for average temperature.

**Figure 1:**
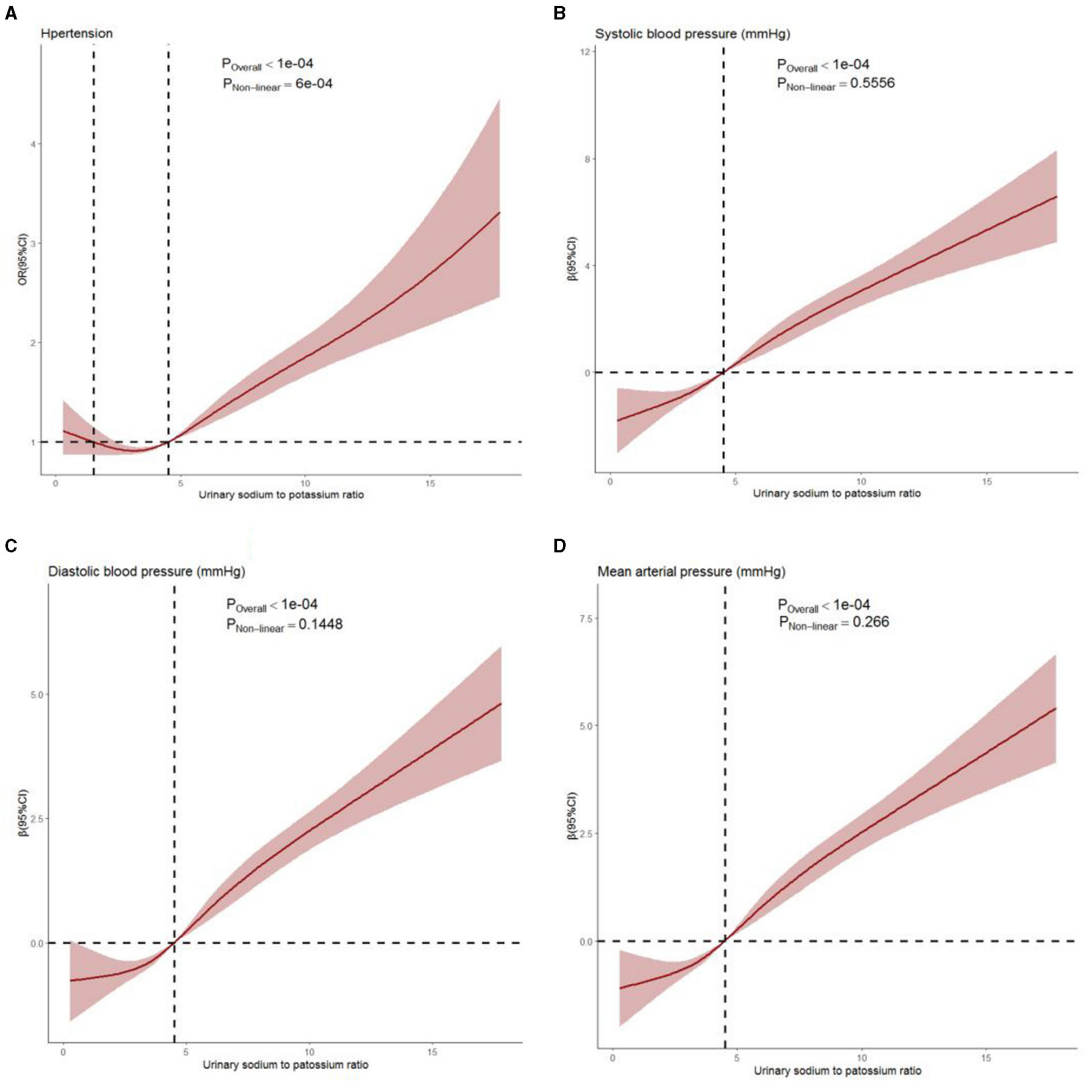
Dose–response relationship between Na/K ratio and hypertension prevalence **(A)**, systolic blood pressure **(B)**, diastolic blood pressure **(C)**, and mean arterial pressure **(D)** levels. Age, gender, region, residence, education, occupation, income level, BMI, regular physical exercise, smoking, alcohol consumption, history of diabetes, history of stroke, history of CHD, eGFR, average temperature, and use of antihypertensive drugs were adjusted. The solid line and shaded region represent the OR or β-value and its 95% confidence intervals (CIs).

Figure 1 shows the dose–response relationship of Na/K with the risk of hypertension and blood pressure levels. There was a non-linear positive association between Na/K and hypertension prevalence (Figure 1A, *P* for non-linearity <0.0001). A linear positive dose–response relationship of Na/K with SBP, DBP, and MAP levels was also observed (Figures 1B–D). The *p*-values for non-linearity were 0.556, 0.145, and 0.266, respectively. The confounding factors adjusted in the RCS models were the same as those adjusted in the logistic or linear regression model 3. As shown in Table 4, the risk of hypertension increased by 9% for each Na/K ratio unit increase, and with each unit increase in Na/K ratio, the SBP, DBP, and MAP increased by 0.53, 0.36, and 0.42 mmHg, respectively. The results changed little after adjusting for average temperature. Moreover, the magnitude of the statistically significant associations with BP indices varied across the different BP groups, except for the fact that the relationship of the Na/K ratio with SBP in participants in the prehypertension group did not reach statistical significance (Table 4). Compared to participants in the normal group and prehypertension group, participants in the hypertension group had a steeper regression slope.

**Table 4:** Associations of Na/K (continuous variable) with the risk of hypertension, SBP, DBP, and MAP levels.

**Outcome**	**Crude^c^**	**Model 1^d^**	**Model 2^e^**	**Model 3^f^**
**Total population**				
Hypertension^a^	1.10 (1.08, 1.11)^*^	1.10 (1.08, 1.11)^*^	1.09 (1.08, 1.11)^*^	1.09 (1.08, 1.11)^*^
SBP^b^	0.77 (0.68, 0.85)^*^	0.64 (0.56, 0.71)^*^	0.54 (0.46, 0.61)^*^	0.53 (0.45, 0.60)^*^
DBP^b^	0.51 (0.46, 0.57)^*^	0.45 (0.39, 0.50)^*^	0.38 (0.33, 0.43)^*^	0.36 (0.31, 0.41)^*^
MBP^b^	0.60 (0.54, 0.66)^*^	0.51 (0.45, 0.57)^*^	0.43 (0.37, 0.49)^*^	0.42 (0.36, 0.47)^*^
**Normotensive group**				
SBP^b^	0.13 (0.07, 0.19)^*^	0.10 (0.04, 0.16)^*^	0.11 (0.05, 0.17)^*^	0.11 (0.05, 0.17)^*^
DBP^b^	0.09 (0.04, 0.14)^*^	0.09 (0.04, 0.14)^*^	0.11 (0.06, 0.15)^*^	0.10 (0.05, 0.15)^*^
MBP^b^	0.11 (0.06, 0.15)^*^	0.09 (0.05, 0.14)^*^	0.11 (0.06, 0.15)^*^	0.10 (0.06, 0.15)^*^
**Prehypertensive group**				
SBP^b^	0.05 (−0.01, 0.11)	0.03 (−0.03, 0.09)	0.04 (−0.02, 0.10)	0.04 (−0.02, 0.10)
DBP^b^	0.10 (0.04, 0.15)^*^	0.11 (0.05, 0.16)^*^	0.10 (0.05, 0.15)^*^	0.09 (0.04, 0.15)^*^
MBP^b^	0.08 (0.04, 0.12)^*^	0.08 (0.04, 0.13)^*^	0.08 (0.04, 0.12)^*^	0.08 (0.03, 0.12)^*^
**Hypertensive group**				
SBP^b^	0.40 (0.22, 0.54)^*^	0.37 (0.21, 0.53)^*^	0.54 (0.38, 0.70)^*^	0.54 (0.38, 0.71)^*^
DBP^b^	0.26 (0.15, 0.36)^*^	0.26 (0.16, 0.36)^*^	0.34 (0.24, 0.44)^*^	0.33 (0.20, 0.43)^*^
MBP^b^	0.30 (0.19, 0.41)^*^	0.29 (0.18, 0.40)^*^	0.40 (0.29, 0.51)^*^	0.40 (0.29, 0.51)^*^

^a^Multivariate logistic regression analysis was performed.

^b^Multivariate linear regression analysis was performed.

^c^Crude model adjusted for none.

^d^Model 1 included adjustment of gender and age.

^e^Model 2 included adjustment of gender, age, region, residence, education, occupation, income level, BMI, regular physical exercise, smoking, alcohol consumption, KABs of dietary sodium intake and hypertension, history of diabetes, history of stroke, history of CHD, eGFR, and use of antihypertensive drugs (only in hypertension group).

^f^Model 3 included model 2 plus adjustment for average temperature. ^*^P < 0.05.

### 3.4 Interaction of gender, age, BMI, smoking, alcohol consumption, regular physical exercise, and spot urinary Na/K ratio on hypertension prevalence and blood pressure indices

We analyzed the relationship of spot urinary Na/K ratio with hypertension and blood pressure levels stratified by gender, age, and BMI (Supplementary Table 2). This association was stronger in female patients and the 50–59 age group. Regarding BMI, the association between the Na/K ratio and hypertension was the strongest among underweight participants, while the associations with blood pressure indices were the strongest in those classified as obese.

**Figure 2:**
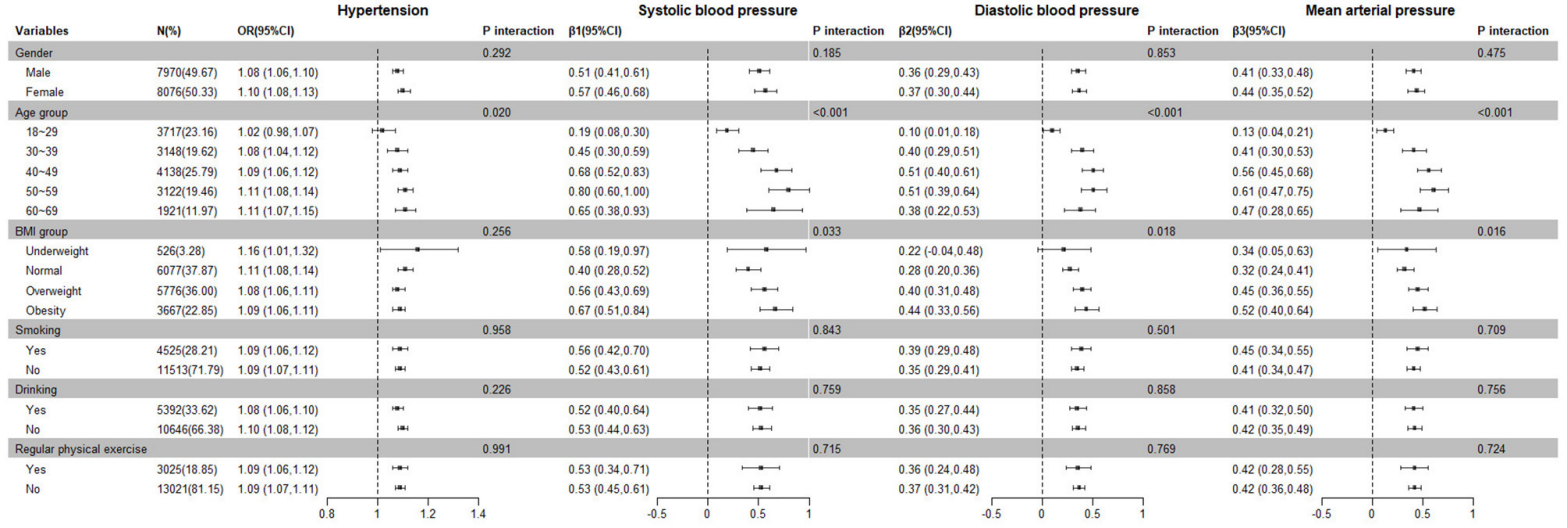
Subgroup and interaction analysis of the relationship between the Na/K ratio and hypertension prevalence and blood pressure levels.

Figure 2 presents the interaction effects of the spot urinary Na/K ratio and other variables on hypertension and blood pressure levels. Age and Na/K ratio had a significant interaction with hypertension. The interactive effect of age and Na/K ratio, BMI, and Na/K ratio were significant on blood pressure levels. There were no significant interactions for gender, smoking, alcohol consumption, regular physical exercise, or the Na/K ratio.

### 3.5 Sensitivity analysis

We used a subsample of participants who had both spot and 24-h urine specimens to conduct the sensitivity analysis. The mean of 24-h urinary sodium and potassium excretions were 176.34 ± 79.08 mmol/day (4.06 ± 1.82 g/day) and 47.26 ± 21.81 mmol/day (1.84 ± 0.85 g/day), respectively. The median 24-h urinary Na/K molar ratio was 3.76 (IQR: 2.68–5.30; Table 1). The Pearson correlation coefficient of spot urinary Na/K and 24-h urinary Na/K was 0.48. Estimates for risk of hypertension by 24-h urine were mostly consistent with those using spot urine (Supplementary Table 3). A stronger effect on blood pressure was found in 24-h urine samples compared to SU (Supplementary Table 3). As shown in Supplementary Figure 1, we found that, when the 24-h urinary Na/K ratio increased, the risk of hypertension and blood pressure increased gradually. The relationships were consistent with those based on SU samples. The results of other sensitivity analyses remained robust (Supplementary Table 4).

## 4 Discussion

In the present study, we found that the urinary Na/K ratio based on spot urine samples was positively associated with the risk of hypertension, SBP, DBP, and MAP. There was also evidence of a dose–response relationship between them, that is, as the urinary Na/K ratio increased, the risk of hypertension and blood pressure levels increased significantly. Our findings also indicate that environmental temperature had little impact on their associations. In addition, sensitivity analysis showed similar associations and dose–response relationships between them when using 24-h urine samples. These findings indicate that spot urinary Na/K was a suitable alternative measurement in population-based epidemiological studies, which is consistent with previous research in diverse populations (19).

Our study demonstrated that one unit of Na/K molar ratio increased SBP and DBP by 0.53 and 0.36 mmHg, respectively, which was consistent with the previous cross-sectional study (1.16 mmHg SBP and 0.84 mmHg DBP increase per 3-units of Na/K) (31). Our findings were also consistent with those of Yin et al., who found that SBP and DBP increased by 4.33 and 1.67 mmHg for a one-unit increase in the gram-to-gram Na/K ratio, respectively (32). Although the strength of the association reported above differed from the results of studies based on 24-h urine samples, the direction of the associations was consistent (22, 23, 25, 27, 33, 34). Furthermore, the use of the Na/K molar ratio (concentration ratios of these two ions) would be more convenient than those conversion values, such as the gram-to-gram ratio of sodium excreted to potassium excreted. It is well-known that accurate estimation of sodium intake can be challenging due to both random and systematic errors. Spot urine samples tend to overestimate or underestimate salt intake at different levels of salt consumption derived from the estimation equations (35, 36). The Na/K ratio has been reported as a surrogate index that is resistant to systematic errors (19). Therefore, spot urinary Na/K is expected to be a useful and convenient indicator in clinical and public health practices, as it is significantly easier to obtain (19).

Several factors, such as gender, age, BMI, renal function, and salt restriction, affect the spot urinary Na/K ratio. We found that female participants, older adults, and those classified as obese had a much stronger association between the Na/K ratio and blood pressure. We additionally found significant interactions between age and Na/K and BMI, and Na/K on blood pressure. The 2018 China Chronic Disease and Risk Factor Surveillance also revealed that BMI played an important role in the relationship between urinary sodium and potassium and blood pressure, although the Na/K ratio showed no significant predominance over sodium or potassium alone (37). The Japanese Nagahama cohort study presented that the association was more significant in older groups (18). The possible reason may be attributed to salt sensitivity. Studies have shown that being female, being of older age, and being obese might increase salt sensitivity of blood pressure (38, 39). Increasing potassium intake might attenuate the frequency and severity of salt sensitivity, particularly in participants whose dietary potassium intake is deficient (40). Therefore, implementing salt reduction strategies should be accompanied by actions to increase potassium intake, especially for those key populations.

The spot urinary Na/K ratio may be influenced by the season of urine collection. Compared with other seasons, the excretion of sodium and potassium in urine decreased in the summer due to elevated excretion via sweat (41). The sodium excreted in sweat has been estimated to be between 0.3 and 2.7 g/L (41). In our study, we were unable to calculate the excretion of sodium through sweat. Therefore, we adjusted for environmental temperature to minimize the bias. However, the temperature had minimal influence on the associations, which may be because our study was conducted during the summer when temperatures did not vary significantly from day to day. Further studies are needed to clarify the impact of sweat or temperature on the associations. We also found that the slope for associations between Na/K ratio and blood pressure was steeper in participants in the hypertension group, which is in line with previous studies. It might be because those in the hypertension group had a higher age and BMI than those in the prehypertension and normal groups. On the other hand, 28–74% of those in the hypertension group are salt sensitive, so they have a high BP response to high sodium intake (38). Participants in the hypertension group will, therefore, have stronger benefits from salt reduction strategies combined with potassium supplementation.

Substantial evidence exists demonstrating the impact of excessive sodium and low potassium intake on the development of hypertension (42, 43). However, few studies have reported the relationship between the spot urinary Na/K ratio and the risk of hypertension in the Chinese general population. Our results showed a positive association between them, with an odds ratio of 1.09 (95% CI: 1.08–1.11) for one unit increment of Na/K molar ratio. In addition, the effect size was larger in the third and fourth quartiles. A cross-sectional study conducted in Dallas also found the same results with an OR of 1.12 (95% CI: 1.02–1.22) for a three-unit increase of Na/K (31). The adjusted OR of hypertension in the highest quartile in our study was 1.78 (95% CI: 1.57–2.01), consistent with findings of a study using 24-h urine samples (OR 1.71, 95% CI: 1.16–2.51) (25). Our findings further confirmed that spot urinary Na/K ratio might be a better predictor of hypertension and elevated blood pressure (18). Future studies are needed to further elucidate its long-term effects and explore whether spot urinary Na/K ratio monitoring can be used as a strategy for blood pressure management.

There are several limitations to the present study. First, as a cross-sectional study, the present study does not elucidate the causality between spot urinary Na/K molar ratio and hypertension. Although we further elucidated the dose–response relationship of Na/K with hypertension prevalence and blood pressure levels, it cannot clarify the long-term effect of spot urinary Na/K on hypertension and blood pressure. Second, we used only a single fasting morning urine specimen for analysis. We found a weak-to-moderate correlation between spot urinary Na/K ratio and 24-h urinary Na/K ratio (r = 0.48), which corroborated the findings of Iwahori et al. (44). They also demonstrated that estimation of the urinary Na/K ratio using six random specimens of daytime casual urine collected on different days served as a good substitute for the traditional 2-day 24-h urine collection. Other studies have also confirmed that, despite a higher correlation between casual Na/K and individual casual sodium or potassium levels, the mean Na/K of 4–7 repeated measurements of casual urine is better for minimizing systematic errors due to diurnal and day-to-day variations (45, 46). The relationship between the mean Na/K of multiple spot urine collections and blood pressure requires further research. Additionally, the current study did not take into account the impact of within-person variability in spot urine sodium-to-potassium ratio, which might obscure the relationship with blood pressure and other health outcomes (47). It also needs to be addressed in future studies by repeated measurements of spot urine samples. Third, spot urinary Na/K is affected by multiple clinical and environmental factors. Therefore, we adjusted as many covariates as possible to minimize the potential effects of confounding factors. We adjusted for renal function using the formula-derived estimates of eGFR, but whether the CKD-EPI equation was appropriate for Chinese was uncertain. Although we adjusted the use of antihypertensive medication, different kinds of antihypertensive drugs could not be assessed. Some evidence suggested that the correlation between spot urinary Na/K and 24-h urinary Na/K was stronger when using calcium channel blockers, angiotensin 2 receptor blockers, and thiazide diuretics (45, 48). This aspect needs to be taken into consideration in future studies.

## 5 Conclusion

This cross-sectional study using data from the SMASH project found that spot urinary Na/K molar ratio had a statistically significant dose–response relationship with hypertension prevalence and blood pressure, and the associations were more pronounced among those with hypertension, female participants, older adults, and those classified as obese. The primary utility of the findings from our study further demonstrates the usefulness of a simple spot urine Na/K ratio as a convenient surrogate index for the more burdensome 24-h urine assessment. Further studies are needed to clarify the long-term effect of spot urinary Na/K on hypertension and blood pressure and address whether it could be used as an intervention indicator in salt reduction action and blood pressure management.

## Data availability statement

The original contributions presented in the study are included in the article/Supplementary material, further inquiries can be directed to the corresponding author.

## Ethics statement

The studies involving humans were approved by the Ethical Committee of the Shandong Center for Disease Control and Prevention. The studies were conducted in accordance with the local legislation and institutional requirements. The participants provided their written informed consent to participate in this study.

## Author contributions

CX: Conceptualization, Investigation, Methodology, Writing – original draft, Writing – review & editing. JD: Methodology, Investigation, Writing – original draft. DL: Methodology, Investigation, Writing – original draft. JX: Project administration, Writing – original draft. BZ: Data curation, Investigation, Writing – original draft. ZL: Data curation, Investigation, Writing – original draft. LW: Project administration, Writing – original draft. JT: Investigation, Writing – original draft. XZ: Project administration, Writing – original draft. JR: Investigation, Writing – original draft. XY: Formal analysis, Writing – original draft. RG: Formal analysis, Writing – original draft. XG: Writing – review & editing, Conceptualization, Methodology, Supervision. JW: Conceptualization, Methodology, Supervision, Writing – review & editing. JM: Conceptualization, Methodology, Supervision, Writing – review & editing.
