# Supplementary material for: Association between spot urinary sodium-to-potassium ratio and blood pressure among Chinese adults aged 18–69 years: the SMASH study
Source: Front Nutr. 2024 Jun 5;11:1383243. doi: 10.3389/fnut.2024.1383243 (PMC11188376; doi:10.3389/fnut.2024.1383243)
Supplement: Supplementary file 1 [file Data_Sheet_1.docx]

Supplementary Material

# **Detailed information about sampling selection of t**he post-intervention survey of the SMASH programme

Shandong Province,situated in the eastern part of China, is the second most populous province with a total population exceeding 95 million in 2016. It is bordered by the Yellow Sea to the east, the Shandong Peninsula to the south, the Yellow River to the west, and the North China Plain to the north.The sample size for the post-intervention survey of the SMASH project was calculated based on a hypertension prevalence estimate of 23.4%, with a margin of error set at 10% of the prevalence estimate (or +/-2.51%) and a 5% type I error. A 90% response rate, 6 stratification factors (geographic locations and economic levels) and a design effect of 2 were considered. The estimated sample size for each stratum is 2796, and the total sample size is 16773. Finally, 16490 participants successfully completed the survey with a participation rate of 96.1%.

# Supplementary Figures and Tables

## Supplementary Tables

**Table S1.** **Distribution of Spot Urinary Na/K ratio in the Studied Population (n=16046)**

| **Variables** | **All (n=16046)** | **Normotensive (n=7597)** | **Prehypertensive (n=4902)** | **Hypertensive (n=3547)** |
| --- | --- | --- | --- | --- |
| **Sex** |  |  |  |  |
| Male | 4.75(3.05-7.04)* | 4.40(2.84-6.57)* | 4.70(3.13-6.91)* | 5.44(3.34-8.01)* |
| Female | 4.31(2.83-6.38) | 4.08(2.67-6.09) | 4.32(2.91-6.34) | 5.09(3.21-7.27) |
| **Age group** |  |  |  |  |
| 18 ~29 | 4.44(2.85-6.78)* | 4.37(2.76-6.69)* | 4.52(3.04-6.99) | 4.83(3.06-7.16) |
| 30~39 | 4.33(2.76-6.44) | 4.07(2.64-6.12) | 4.56(2.94-6.74) | 5.14(3.23-7.33) |
| 40~49 | 4.48(2.95-6.69) | 4.06(2.68-6.07) | 4.48(3.05-6.67) | 5.38(3.49-7.71) |
| 50~59 | 4.64(3.04-6.78) | 4.12(2.87-5.99) | 4.56(3.10-6.42) | 5.34(3.19-7.72) |
| 60~69 | 4.81(3.15-6.86) | 4.36(3.02-6.20) | 4.72(3.01-6.47) | 5.14(3.29-7.72) |
| **Residence** |  |  |  |  |
| Urban | 4.53(3.01-6.65) | 4.17(2.81-6.24) | 4.62(3.17-6.70) | 5.33(3.37-7.62) |
| Rural | 4.49(2.90-6.73) | 4.20(2.71-6.28) | 4.51(2.97-6.65) | 5.21(3.23-7.66) |
| **Region** |  |  |  |  |
| East | 4.05(2.61-6.08)* | 3.74(2.40-5.76)* | 4.14(2.84-6.08)* | 4.49(2.80-6.51)* |
| Middle and South | 4.27(2.82-6.39) | 4.05(2.69-5.99) | 4.37(2.89-6.42) | 5.03(3.08-7.24) |
| Northwest | 5.10(3.33-7.44) | 4.63(3.05-6.76) | 5.24(3.43-7.63) | 6.15(4.12-8.87) |
| **Education** |  |  |  |  |
| Primary, middle school, and under | 4.58(3.00-6.81)* | 4.24(2.82-6.34)* | 4.60(3.06-6.67) | 5.33(3.33-7.76)* |
| High school and above | 4.30(2.76-6.46) | 4.09(2.60-6.06) | 4.35(2.94-6.62) | 5.01(3.07-7.13) |
| **Occupation** |  |  |  |  |
| (Hard physical work) Farmer/Peasant/Manual worker | 4.51(2.93-6.77)* | 4.13(2.71-6.20) | 4.54(3.01-6.78) | 5.30(3.31-7.70) |
| (Light physical work) Service/Administrative/Technical/Professionals/Others | 4.51(2.95-6.67) | 4.30(2.81-6.35) | 4.54(3.11-6.62) | 5.35(3.27-7.71) |
| Underemployment/Retired | 4.34(2.80-6.15) | 3.96(2.58-5.91) | 4.44(2.87-5.88) | 4.89(3.08-6.80) |
| **Annual household income** |  |  |  |  |
| First tertile | 4.58(2.97-6.72) | 4.21(2.77-6.25) | 4.65(3.11-6.63) | 5.23(3.24-7.67) |
| Second tertile | 4.48(2.91-6.71) | 4.14(2.75-6.17) | 4.55(2.97-6.79) | 5.27(3.26-7.68) |
| Third tertile | 4.42(2.91-6.63) | 4.18(2.73-6.30) | 4.41(2.99-6.61) | 5.22(3.29-7.40) |
| **Lifestyle status** |  |  |  |  |
| **Smoking** |  |  |  |  |
| Yes | 4.76(3.09-7.03)* | 4.36(2.83-6.53)* | 4.73(3.14-6.87)* | 5.57(3.52-8.01)* |
| No | 4.40(2.88-6.57) | 4.13(2.70-6.19) | 4.46(2.97-6.57) | 5.11(3.18-7.45) |
| **Drinking** |  |  |  |  |
| Yes | 4.82(3.13-7.08)* | 4.46(2.91-6.61)* | 4.75(3.22-6.98)* | 5.42(3.35-7.98)* |
| No | 4.36(2.83-6.52) | 4.10(2.65-6.13) | 4.40(2.89-6.5) | 5.13(3.21-7.42) |
| **Regular physical exercise** |  |  |  |  |
| Yes | 4.39(2.84-6.50)* | 4.04(2.64-5.98)* | 4.37(2.93-6.50) | 5.14(3.06-7.35) |
| No | 4.52(2.95-6.75) | 4.23(2.76-6.33) | 4.57(3.06-6.70) | 5.28(3.34-7.68) |
| **Disease status** |  |  |  |  |
| **Diabetes** |  |  |  |  |
| Yes | 4.53(2.95-6.74) | 3.97(2.62-5.75) | 4.35(3.01-6.20) | 5.08(3.04-7.30) |
| No | 4.50(2.93-6.70) | 4.20(2.75-6.28) | 4.57(3.04-6.71) | 5.28(3.33-7.73) |
| **Stroke** |  |  |  |  |
| Yes | 5.38(3.22-8.27)* | 5.17(3.22-5.69) | 4.46(2.94-6.62) | 5.73(3.45-10.08)* |
| **No** | 4.50(2.93-6.69) | 4.18(2.74-6.27) | 4.53(3.04-6.66) | 5.25(3.27-7.58) |
| **Coronary heart disease** |  |  |  |  |
| Yes | 4.84(2.99-6.86) | 4.24(3.07-6.25) | 5.13(3.38-6.88) | 4.93(2.82-7.34) |
| No | 4.50(2.93-6.70) | 4.19(2.74-6.26) | 4.52(3.04-6.65) | 5.29(3.31-7.68) |
| **BMI status** |  |  |  |  |
| Underweight | 4.25(2.64-6.43)* | 4.05(2.59-6.19) | 4.72(2.72-6.68) | 5.39(4.07-7.24) |
| Normal | 4.37(2.85-6.50) | 4.22(2.74-6.28) | 4.51(3.02-6.56) | 5.26(3.28-7.61) |
| Overweight | 4.51(2.97-6.69) | 4.17(2.79-6.21) | 4.51(3.06-6.71) | 5.14(3.28-7.47) |
| Obesity | 4.80(3.05-7.08) | 4.27(2.71-6.49) | 4.65(3.06-6.74) | 5.38(3.27-7.78) |
| **KABs of salt and hypertension** |  |  |  |  |
| As salt intake decreases, do does blood pressure |  |  |  |  |
| Yes | 4.44(2.88-6.62)* | 4.11(2.67-6.16)* | 4.48(3.00-6.60)* | 5.17(3.19-7.51)* |
| No | 4.66(3.05-6.89) | 4.36(2.87-6.44) | 4.71(3.10-6.85) | 5.45(3.48-8.02) |
| Approval of low salt diet |  |  |  |  |
| Yes | 4.48(2.93-6.66)* | 4.16(2.74-6.23)* | 4.52(3.02-6.62) | 5.26(3.29-7.60) |
| No | 4.75(3.06-7.13) | 4.59(2.84-6.66) | 4.70(3.2-7.36) | 5.28(3.11-8.01) |
| Have taken action to reduce dietary salt intake |  |  |  |  |
| Yes | 4.40(2.90-6.54)* | 4.09(2.70-6.07)* | 4.46(3.00-6.5)* | 5.13(3.19-7.47)* |
| No | 4.69(3.01-7.01) | 4.38(2.81-6.56) | 4.67(3.10-7.01) | 5.51(3.51-8.00) |
| Sodium/potassium ratio category |  |  |  |  |
| <2.0 | 1.47(1.11-1.75)* | 1.48(1.12-1.76)* | 1.44(1.06-1.75)* | 1.48(1.12-1.77)* |
| 2.0~3.0 | 2.52(2.27-2.76) | 2.52(2.28-2.76) | 2.53(2.28-2.78) | 2.49(2.23-2.76) |
| >3.0 | 5.52(4.14-7.59) | 5.26(4.02-7.21) | 5.45(4.13-7.47) | 6.11(4.54-8.52) |
| Estimated sodium excretion category |  |  |  |  |
| <3.0 | 2.26(1.48-3.25)* | 2.35(1.58-3.29)* | 2.26(1.44-3.29)* | 1.94(1.27-2.91)* |
| 3.0~5.0 | 4.79(3.35-6.71) | 4.71(3.33-6.66) | 4.79(3.36-6.67) | 5.00(3.37-6.84) |
| >5.0 | 7.93(5.74-10.57) | 7.15(5.04-9.70) | 7.64(5.41-10.09) | 8.79(6.51-11.47) |
| Estimated potassium excretion category |  |  |  |  |
| <1.9 | 4.78(3.17-7.00)* | 4.41(2.95-6.51)* | 4.84(3.29-6.98)* | 5.60(3.67-8.11)* |
| 1.9~2.5 | 2.66(1.80-3.89) | 2.43(1.66-3.41) | 2.72(1.86-3.96) | 3.04(2.01-4.67) |
| >2.5 | 2.02(1.28-2.87) | 2.09(1.43-2.85) | 1.62(1.20-2.47) | 2.32(1.61-3.17) |
| eGFR category |  |  |  |  |
| ≥90 | 4.50(2.94-6.7) | 4.21(2.75-6.28) | 4.54(3.07-6.74) | 5.29(3.32-7.63) |
| 60~89 | 4.53(2.9-6.74) | 4.01(2.73-5.89) | 4.42(2.86-6.29) | 5.18(3.11-7.68) |
| 30~59 | 4.80(2.57-6.44) | 3.64(3.64-3.64) | 4.84(3.35-5.44) | 4.80(2.37-6.59) |
| <30 | 4.09(2.46-5.04) | 4.09(2.36-4.88) | 4.20(2.46-7.94) | 3.05(3.04-3.10) |

Data are IQR or mean ± standard deviation.BMI: body mass index; SBP: systolic blood pressure; DBP: diastolic blood pressure; eGFR: estimated glomerular filtration rate. ^*^ *P*<0.05.

**Table S2.** **Associations of Na/K ratio with hypertension prevalence, SBP, DBP and MAP levels stratified by gender, age group and BMI**

| Variables | Hypertension | SBP | DBP | MAP |
| --- | --- | --- | --- | --- |
| Gender |  |  |  |  |
| Male | 1.08(1.06,1.10)^*^ | 0.51(0.41,0.61)^*^ | 0.36(0.29,0.43)^*^ | 0.41(0.33,0.48)^*^ |
| Female | 1.10(1.08,1.13)^*^ | 0.57(0.46,0.68)^*^ | 0.37(0.30,0.44)^*^ | 0.44(0.35,0.52)^*^ |
| Age group |  |  |  |  |
| 18~29 | 1.02(0.98,1.07)^*^ | 0.19(0.08,0.30)^*^ | 0.10(0.01,0.18)^*^ | 0.13(0.04,0.21)^*^ |
| 30~39 | 1.08(1.04,1.12)^*^ | 0.45(0.30,0.59)^*^ | 0.40(0.29,0.51)^*^ | 0.41(0.30,0.53)^*^ |
| 40~49 | 1.09(1.06,1.12)^*^ | 0.68(0.52,0.83)^*^ | 0.51(0.40,0.61)^*^ | 0.56(0.45,0.68)^*^ |
| 50~59 | 1.11(1.08,1.14)^*^ | 0.80(0.60,1.00)^*^ | 0.51(0.39,0.64)^*^ | 0.61(0.47,0.75)^*^ |
| 60~69 | 1.11(1.07,1.15)^*^ | 0.65(0.38,0.93)^*^ | 0.38(0.22,0.53)^*^ | 0.47(0.28,0.65)^*^ |
| BMI group |  |  |  |  |
| Underweight | 1.16(1.01,1.32)^*^ | 0.58(0.19,0.97)^*^ | 0.22(-0.04,0.48) | 0.34(0.05,0.63)^*^ |
| Normal | 1.11(1.08,1.14)^*^ | 0.40(0.28,0.52)^*^ | 0.28(0.20,0.36)^*^ | 0.32(0.24,0.41)^*^ |
| Overweight | 1.08(1.06,1.11)^*^ | 0.56(0.43,0.69)^*^ | 0.40(0.31,0.48)^*^ | 0.45(0.36,0.55)^*^ |
| Obesity | 1.09(1.06,1.11)^*^ | 0.67(0.51,0.84)^*^ | 0.44(0.33,0.56)^*^ | 0.52(0.40,0.64)^*^ |

Data are odds ratio (OR) or regression coefficient ( β ) and its 95% CI,^*^ *P*<0.05. Gender, age, region, residence, education, occupation, income level, BMI, regular physical exercise, smoke, drinking, diabetes history, stroke history, CHD history, eGFR or anti-hypertensive drug use and average temperature were adjusted.

**Table S3. Associations of Na/K ratio with the risk of Hypertension, SBP, DBP and MAP levels (n=1671)**

| **Outcome** | Crude^a^ | Model 2^b^ | Model 3^c^ | Model 4^d^ |
| --- | --- | --- | --- | --- |
| **24h urine** |  |  |  |  |
| Hypertension^1^ | 1.11(1.06,1.16) | 1.18(1.12,1.24) | 1.15(1.09,1.22) | 1.15(1.08,1.22) |
| SBP^2^ | 0.93(0.55,1.30) | 1.09(0.74,1.45) | 0.82(0.47,1.17) | 0.79(0.44,1.14) |
| DBP^2^ | 0.72(0.48,0.97) | 0.81(0.56,1.05) | 0.62(0.39,0.85) | 0.59(0.36,0.81) |
| MAP^2^ | 0.79(0.52,1.07) | 0.90(0.64,1.17) | 0.69(0.43,0.94) | 0.65(0.40,0.91) |
| **Spot urine** |  |  |  |  |
| Hypertension^1^ | 1.12(1.09,1.15) | 1.13(1.09,1.17) | 1.13(1.09,1.17) | 1.12(1.08,1.17) |
| SBP^2^ | 0.89(0.65,1.13) | 0.78(0.55,1.01) | 0.62(0.40,0.84) | 0.59(0.40,0.81) |
| DBP^2^ | 0.65(0.50,0.81) | 0.60(0.45,0.78) | 0.51(0.37,0.66) | 0.48(0.33,0.63) |
| MAP^2^ | 0.73(0.55,0.91) | 0.66(0.49,0.83) | 0.55(0.39,0.71) | 0.52(0.35,0.68) |

Data are odds ratio (OR) or regression coefficient ( β ) and its 95% CI, *P*<0.05.. ^1^Multivariate logistic regression analysis was performed;^2^Multivariate linear regression analysis was performed; ^a^ Crude model adjusted for none; ^b^ Model 1 included adjustment of gender and age; ^c^ Model 2 included adjustment of gender, age, region, residence, education, occupation, income level, BMI, regular physical exercise, smoke, drinking, diabetes history, stroke history, CHD history, eGFR and anti-hypertensive drug use; ^d^ Model 3: model 2 plus adjustment for average temperature. SBP: systolic blood pressure; DBP: diastolic blood pressure; MAP: mean arterial pressure.

**Table S4. Results of sensitivity analyses among the Studied Population**

| **Characteristics** | **Hypertension^1^** | **SBP^2^** | **DBP^2^** | **MBP^2^** |
| --- | --- | --- | --- | --- |
| (1) Individuals without taking antihypertension medicine (n=14619) | | | | |
|  | 1.08(1.06,1.10)* | 0.52(0.44,0.60)* | 0.36(0.31,0.42)* | 0.42(0.36,0.47)* |
| (2) Individuals without stroke and coronary heart diseases (n=15476) | | | | |
|  | 1.09(1.08,1.11)* | 0.52(0.44,0.60)* | 0.36(0.30,0.41)* | 0.41(0.35,0.47)* |
| (3) Excluding alcohol use from covariates (n=16046) | | | | |
|  | 1.09(1.08,1.11)* | 0.53(0.46,0.61)* | 0.37(0.32,0.42)* | 0.42(0.37,0.48)* |
| (4) Excluding regular physical exercise from covariates (n=16046) | | | | |
|  | 1.09(1.07,1.11)* | 0.53(0.45,0.60)* | 0.37(0.31,0.41)* | 0.42(0.36,0.47)* |
| (5) Excluding occupation and income levels from covariates (n=16046) | | | | |
|  | 1.09(1.08,1.11)* | 0.52(0.45,0.60)* | 0.36(0.31,0.41)* | 0.41(0.36,0.47)* |
| (6) Excluding KABs of salt and hypertension from covariates (n=16046) | | | | |
|  | 1.09(1.07,1.10)* | 0.53(0.45,0.60)* | 0.36(0.31,0.41)* | 0.41(0.36,0.47)* |

Data are odds ratio (OR) or regression coefficient ( β ) and its 95% CI, *P*<0.05.. ^1^Multivariate logistic regression analysis was performed;^2^Multivariate linear regression analysis was performed; SBP: systolic blood pressure; DBP: diastolic blood pressure; MAP: mean arterial pressure.

## Supplementary Figures

| 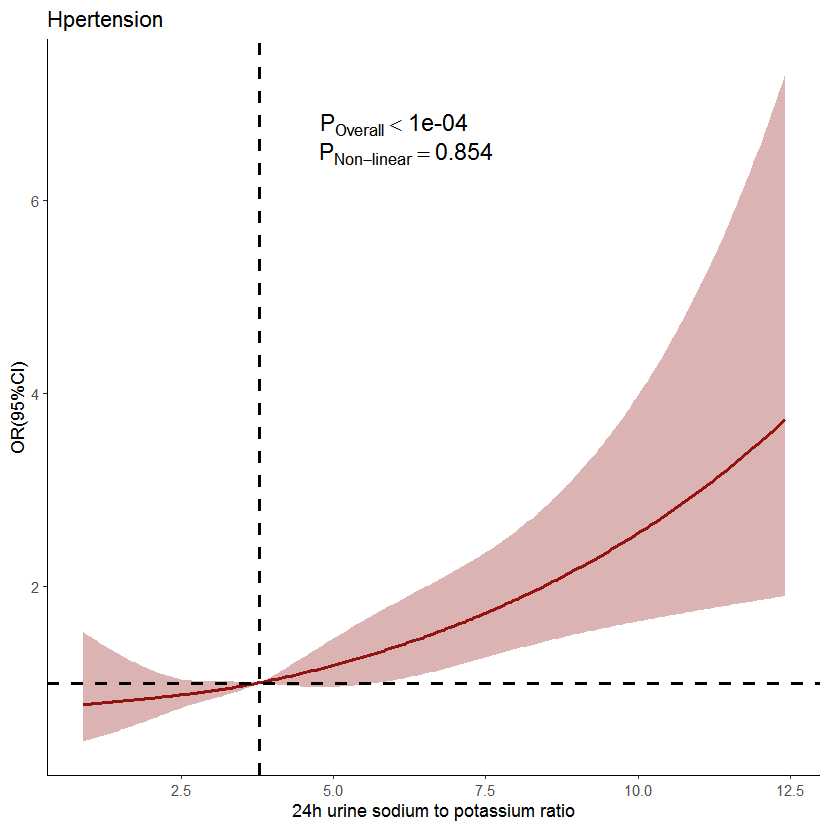 | 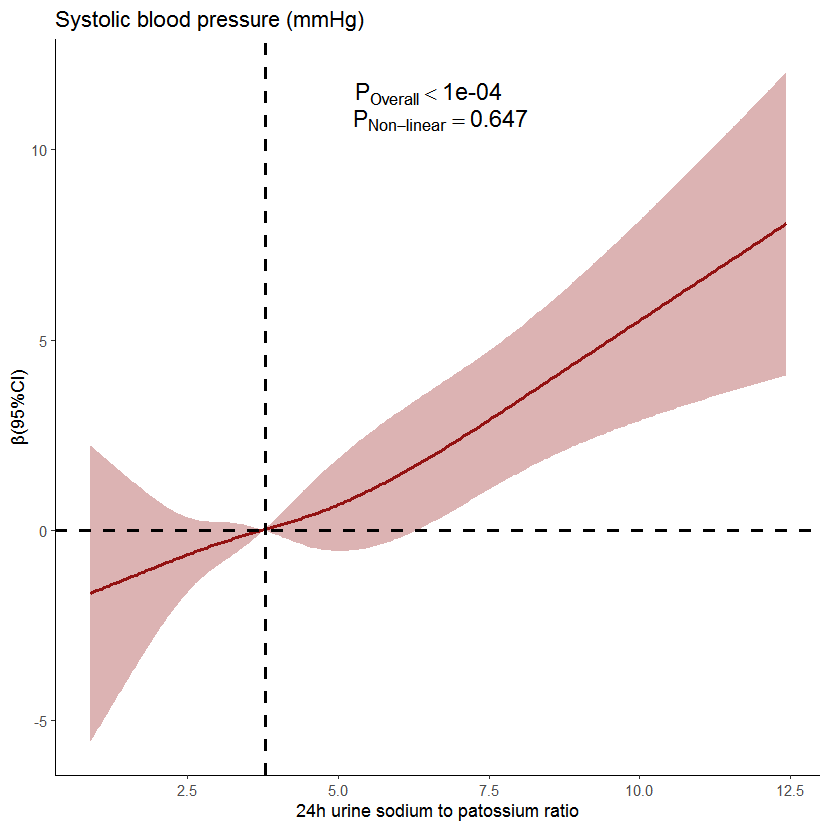 |
| --- | --- |
| (**a**) | (**b**) |
| 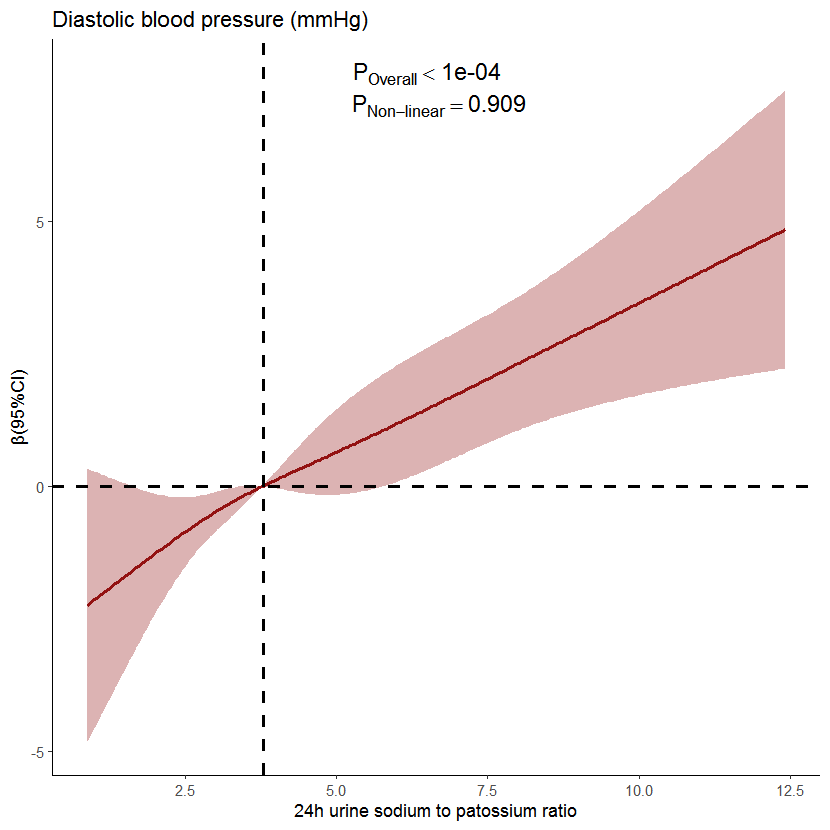 | 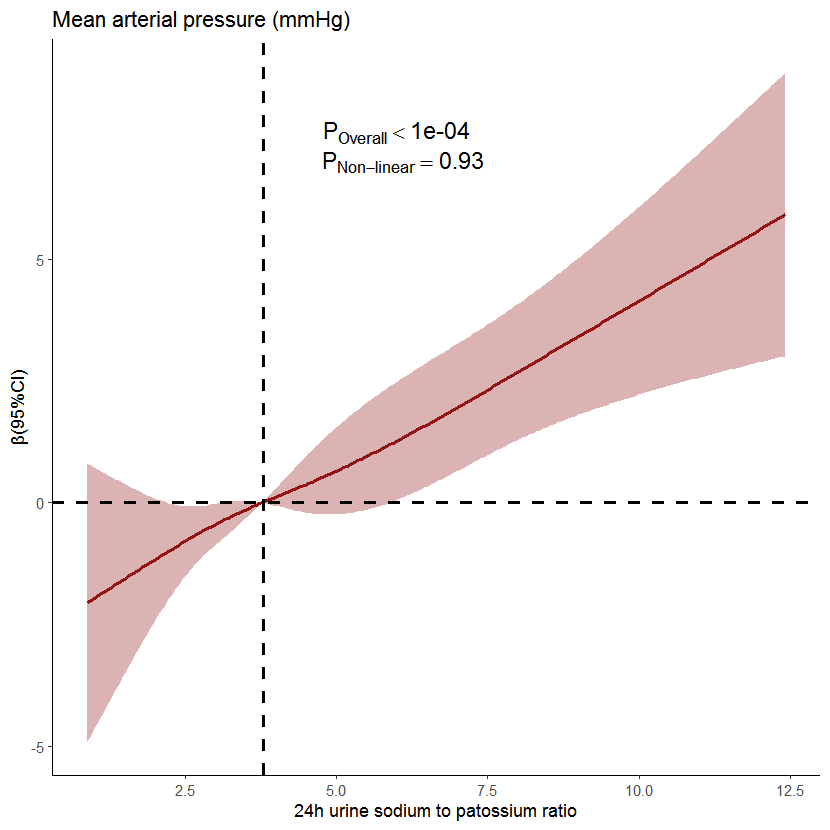 |
| (**c**) | (**d**) |

**Figure S1.** Dose-response relationship of 24-hour urinary sodium to potassium ratio and hypertension prevalence (**a**), systolic blood pressure (**b**), diastolic blood pressure (**c**) and mean arterial pressure (**d**) levels. Age, gender, region, residence, education, occupation, income level, BMI, regular physical exercise, smoke, drinking, diabetes history, stroke history, CHD history, eGFR, average temperature and anti-hypertensive drug use were adjusted. The solid line and shaded region represent OR or β and its 95% confidence intervals.
